# Mission imputable: Effects of missing data processing on infectious disease detection and prognosis

**DOI:** 10.1371/journal.pone.0320105

**Published:** 2026-07-27

**Authors:** Suravi Saha Roy, Ngoc Thi Nguyen, Agustin Zuniga, Fatemeh Sarhaddi, Eemil Lagerspetz, Huber Flores, Petteri Nurmi

**Affiliations:** 1 Department of Computer Science, University of Helsinki, Helsinki, Finland; 2 Department of Computer Science, University of Tartu, Tartu, Estonia; Firat Universitesi, TÜRKIYE

## Abstract

**Background:**

Missing data in medical datasets poses significant challenges for developing effective AI/ML pipelines. Inaccurate imputation can lead to biased results, reduced model performance, and compromised clinical insights. Understanding how different imputation methods affect AI/ML model performance is crucial for ensuring accurate clinical findings.

**Objective:**

This study systematically investigates the effects of different imputation methods on AI/ML model performance and their clinical implications.

**Methods:**

We investigate the impact of six different missing data strategies on the performance of common classification algorithms for analyzing medical data. The performance was evaluated based on sensitivity and specificity metrics for the tasks of predicting COVID-19 diagnosis and patient deterioration. We also perform feature analysis to understand the clinical implications that the choice of imputation method has.

**Results:**

The findings reveal that the effects of imputation depend on the clinical setting. For a general screening cohort (Einstein Data4u), multivariate imputation by chained equations (MICE) yielded the best performance in clinical settings, resulting in a 26% improvement in sensitivity compared to baseline methods and unmasking critical viral coinfections. Conversely, in an intensive care cohort (MIMIC-IV), complete-case analysis initially showed higher raw predictive metrics. However, further analysis demonstrates that this stems from selection bias driven by informative missingness (MNAR), where testing patterns are intrinsically tied to patient severity. Thus, while imputation recovers diagnostic signals in sparse screening data, it serves as a crucial tool for reducing bias in high-acuity settings.

**Conclusion:**

This study demonstrates the critical impact of missing data imputation on AI/ML model performance and the resulting clinical insights. Our findings underscore the importance of selecting appropriate imputation techniques tailored to the specific characteristics of medical data to ensure accurate and reliable AI/ML predictions. By utilizing a rigorous cross-validation pipeline and a systematic comparison, we provide insights for selecting the most appropriate imputation methods for clinical decision-making applications.

## Introduction

Artificial Intelligence (AI) and Machine Learning (ML) methods have significant potential to alleviate pressure on healthcare systems by offering cost-effective and efficient solutions for supporting the analysis, detection, and care of health conditions. Indeed, these techniques have demonstrated considerable promise in enhancing the diagnosis of various conditions, including cancer [1], cardiovascular diseases [2], lung infections [3], and diabetes onset [4]. However, the development of effective AI/ML pipelines is often challenged by the heterogeneous nature of medical data, which is frequently characterized by high sparsity and substantial amounts of missing data [5]. If not adequately addressed, these data quality issues can significantly compromise the accuracy of predictive models and the validity of clinical insights derived from them. For instance, Cismondi et al. [6] demonstrated an 11% difference in accuracy for detecting septic shocks based solely on whether missing data were appropriately handled or omitted.

Typically, methods for handling missing data are tailored to individual studies, resulting in limited consensus regarding best practices. Recent research has explored various imputation methods [7] for both general datasets and health-specific datasets [8]. Other studies have reviewed imputation techniques applied to healthcare applications based on clinical datasets for various diseases [9,10]. While these works provide valuable overviews of imputation methods across several healthcare domains, comprehensive and systematic evaluations of these techniques, particularly for infectious diseases, remain lacking. A detailed understanding of the advantages and disadvantages of different procedures for handling missing data, as well as their impact on the performance of AI and ML methods, is essential for developing reusable pipelines that produce reliable clinical insights.

The massive scale of the COVID-19 pandemic highlighted the importance of high-throughput AI/ML applications in supporting healthcare and reducing the burden of health professionals during infectious disease outbreaks. The use of AI/ML to detect COVID-19 infections, or to predict potential patient deterioration, are two representative examples where automated analysis of medical data can be highly valuable. Consequently, since the onset of the COVID-19 pandemic, there has been a significant interest in developing methods to predict COVID-19 risks, infections, and outcomes [11]. These efforts range from analyses using chest X-ray images [12] to studies leveraging laboratory tests [13,14] or electronic health records [15]. While promising, studies have been highly customized without offering insights into the effects of imputation techniques on AI/ML pipelines or the clinical insights drawn from these analyses. Indeed, practically all existing studies are affected by significant amounts of missing data and high data sparsity, yet the methods for handling missing data are largely determined on a case-by-case basis, often without a comprehensive understanding of their clinical effects.

This paper contributes a comprehensive and systematic evaluation of missing data imputation accuracy within healthcare data, analyzing the subsequent effects on AI/ML performance, with a particular focus on the clinical implications of these methods. We investigate six distinct strategies, which comprise five imputation methods and a no-imputation baseline, integrated into a cost-effective pipeline for COVID-19 diagnosis and prognosis. This methodological evaluation facilitates the early detection and the prediction of patient deterioration, addressing the dual needs of high diagnostic accuracy and operational scalability in data-constrained clinical settings. Our analysis utilizes two real-world datasets to ensure generalizability: (i) the openly available Hospital Israelita Albert Einstein dataset [16], which contains medical records from 1591 patients, and (ii) the MIMIC-IV dataset [17], a large-scale critical care database. Each record contains values for laboratory tests, diagnosis status, and information about possible patient admissions to semi-intensive (high-dependency) or intensive care units.

Our results demonstrate that model performance is heavily influenced by how missing data is handled, with accuracy improvements of up to 26 percentage points achievable through the incorporation of appropriate imputation techniques. The optimal approach depends on the characteristics of the missing data itself, including feature type, frequency, and pattern. Generally, ignoring missing data (i.e., no imputation) results in the poorest performance, while Multiple Imputation by Chained Equations (MICE) offers the best outcomes in most scenarios. We also analyze the importance of different features and highlight how the most significant predictors vary depending on the data processing method. For instance, when no imputation is performed, patient age quantile emerges as the best predictor for patient deterioration. However, viral and blood test features become more critical once the data is imputed. This demonstrates that imputation not only affects the performance of algorithms but can also alter clinical interpretations. By optimizing this pipeline, we achieved 81% sensitivity and 98% specificity for early screening of COVID-19 positive patients, and 65% sensitivity and 99% specificity for predicting patient deterioration.

Our work offers novel insights into the effects of imputation techniques, demonstrating the importance of missing data processing in the design of effective AI/ML pipelines. Researchers and data scientists can leverage our findings to improve the design of robust AI/ML pipelines, while clinicians are provided with a validated method for the early screening of suspected disease cases and the optimization of triage protocols. By acting as a secondary diagnostic tool using low-cost, readily available laboratory test results, our approach assists in the prioritization of patient care and the efficient allocation of critical medical resources, offering deeper insights into the factors driving model performance.

## Methods

### Datasets

We utilize two real-world medical datasets to ensure the validity of our findings. The use of these real-world medical datasets ensures that our data is representative of regular clinical practices and incorporates clinical data from laboratory tests on suspected COVID-19 patients. This allows us to capture the relationships between the laboratory test outcomes, COVID-19 diagnosis, and patient deterioration.

**Einstein Data4u**: As our first dataset, we used an open-source dataset made publicly available by the Hospital Israelita Albert Einstein in São Paulo, Brazil, through Kaggle [16]. The dataset was anonymized by the publisher in accordance with international privacy guidelines, ensuring that no personally identifiable information is accessible. Therefore, additional ethical approval was not required for its use in this research. The data were accessed and downloaded in April 2022 for the development of a COVID-19 screening and triage pipeline. This dataset was utilized to evaluate the influence of missing data imputation methods. In total, 1591 patient records were used in this study, which consist of anonymized data from patients tested for COVID-19 using RT-PCR (positive or negative), admission decisions for positive cases (regular ward, semi-intensive (high-dependency) or intensive care unit), and 106 laboratory tests collected during their visit to the emergency room. The data are characterized by a significant skew in outputs (approximately 9% of the samples are positive for COVID-19 tests) and a large number of missing laboratory test values, with 77.25% of the samples missing at least one laboratory test result. The dataset comprised 5644 records. A significant proportion (71.81%; *n* = 4053) lacked values for the 20 specific laboratory tests selected for this analysis. These records were excluded from our study to ensure a consistent feature matrix for the evaluation of imputation strategies.

**MIMIC-IV v3.1 [17]:** Medical Information Mart for Intensive Care (MIMIC)-IV is a large and well-established open dataset of patients in emergency or intensive care. This dataset contains de-identified data of over 265,000 patients between 2008−2022. For privacy purposes, the admission time of the patients has been shifted. The patient data include 381 lab tests, ICU stay information, and diagnoses, but there is no COVID-19 test in the features. Therefore, we only considered the data of patients who had been diagnosed with COVID-19. The dataset contains 3,620 patients diagnosed with COVID-19. Some patients (665) were diagnosed with COVID-19 more than once; however, we only considered the first record for each patient in this study to reduce potential correlation between the records. We also removed patient records that had no values for any of the laboratory tests considered in the analysis. After excluding these patient records, the final MIMIC-IV dataset contained 3,337 samples.

### Data analysis

**Fig 1:**
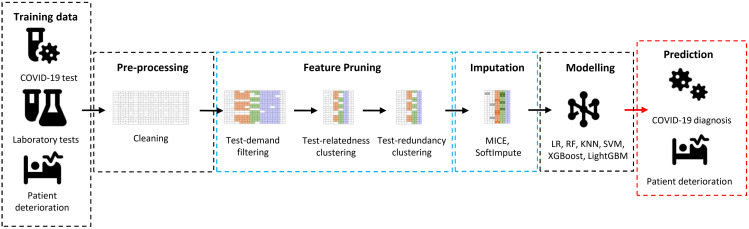
Missing data imputation pipeline through feature pruning and missing value imputation.

The AI/ML pipeline for COVID-19 screening, triage, and the comparative analysis of imputation methods is illustrated in Fig 1. The pipeline consists of four stages: pre-processing, pruning, imputation, and modeling, allowing a systematic assessment of predictive integrity across different imputation strategies. In the pre-processing step, input data, including COVID-19 tests, laboratory tests, and patient deterioration data, are cleaned to remove empty records and to transform the features into numerical values. In the feature pruning step, the dimensionality of the data is reduced by selecting representative features and removing dependent ones. This process was tailored to the specific characteristics of each dataset (see Section Feature Analysis). The imputation step fills in the missing data by applying one of the techniques considered in the study: no imputation (baseline), single imputation, multiple imputation (KNN, MICE), Matrix factorization–based imputation (SoftImpute), or Random forest–based imputation (MissForest). Finally, in the modeling step, different classifiers are trained and evaluated to predict COVID-19 diagnosis and patient deterioration. The classifiers include logistic regression (LR), random forest (RF), support vector machine (SVM), k-nearest neighbors (KNN), XGBoost, and LightGBM. These steps are described in detail in the following sections.

### Feature analysis

Medical practices and procedures govern which medical tests and procedures a specific patient undergoes. As these vary among patients, there are certain commonalities but also variations across patients. Accounting for these variations in procedures can be used to alleviate data sparsity. Specifically, the availability of a particular test variable depends on three interlinked factors: (i) *demand* for specific test procedures, i.e., whether a patient is required to undergo a specific laboratory test; (ii) *relatedness*, i.e., several tests monitor factors that are closely correlated with another test and it may suffice to use one of the tests; and (iii) *redundancy*, i.e., tests may measure the same aspect and be candidates for replacement. We take advantage of these three criteria to reduce the feature space and minimize biases and inaccuracies in the analysis.

**Feature selection.** In this step, we first removed the features with a large number of missing values. These features impose higher complexity for imputation and increase the sparsity of the modeling. Most of these removed features are missing completely at random (MCAR) or the underlying reason for missing values is difficult to deduce (i.e., MNAR) and have over 90% of values missing without any distinguishable patterns. In total, 55 continuous and 18 discrete features were removed from the Data4u dataset. For the MIMIC-IV dataset, 281 continuous features were excluded, ensuring that only the most significant and independent laboratory markers were retained for analysis.

For the Data4u dataset, the 33 retained features are categorized as follows: (i) 14 hematological parameters (complete blood count), which have demonstrated efficacy in detecting COVID-19 during the second week of symptom onset [18], alongside rapid antigen tests for influenza A and B; (ii) 17 variables representing various viral pathogen families; and (iii) the COVID-19 test result and subsequent hospital admission decision. Pathogens belonging to identical families were combined into unified features to reduce the dimensionality of the input space. Based on the assumption that pathogen absence reflects a non-infected state, we utilized binary encoding to represent each family. A value of 1 was assigned if at least one test within the family yielded a positive result, and 0 if all constituent tests were negative. This domain-driven aggregation produced the following six features:

1. *Adenoviridae:* Adenovirus
2. *Coronaviridae:* Coronavirusnl63, Coronavirus hku1, Coronavirus229e and Coronavirusoc43
3. *Orthomyxoviridae:* Influenza a, Influenza b and Inf a h1n1 2009
4. *Paramyxoviridae:* Parainfluenza 1, Parainfluenza 2, Parainfluenza 3 and Parainfluenza 4
5. *Picornaviridae:* Rhinovirus/Enterovirus
6. *Pneumoviridae:* Chlamydophila pneumoniae, Metapneumovirus and Respiratory syncytial virus

Two additional rapid test features were removed from the feature space as they are already included in the Orthomyxoviridae virus family. Finally, we removed hematocrit from the feature space as it has a high correlation with hemoglobin [19]. Since both parameters reflect red blood mass, using only one of these features suffices to avoid multicollinearity and clinical redundancy. We retained hemoglobin as it demonstrated higher data completeness within the cohort. The selected features for Data4u are shown in Table 1.

**Table 1:** Einstein Data4u Dataset construction.

Tasks	Samples	Without missing values	With missing values	Predicted features	Numerical features (13)	Categorical features (8)
Task 1	Positive	50	143	COVID-19 diagnosis	MCHC, MCV, MCH, RDW, Red blood cells, Eosinophils, Mean platelet volume, Lymphocytes, Platelets, Hemoglobin, Leukocytes, Basophils, Monocytes.	Coronaviridae, Orthomyxoviridae, Paramyxoviridae, Picornaviridae, Pneumoviridae, Adenoviridae, Bordetella pertussis, Patient age quantile
	Negative	312	1448			
	**Total**	**362**	**1591**			
Task 2	Deteriorate	56	89	COVID-19 patient deterioration		
	No-deteriorate	306	1502			
	**Total**	**362**	**1591**			

For the MIMIC-IV dataset, we retained a feature space consistent with the Data4u dataset, with the exception of viral pathogen family indicators, which were unavailable in this cohort. We augmented the feature set with additional clinical variables previously validated for coronavirus mortality prediction within the MIMIC data [20]. Table 2 shows the selected features of the MIMIC-IV dataset.

**Table 2:** MIMIC-IV extracted dataset construction.

Task	Class	Without missing values	With missing values	Predicted features	Numerical features (28)
Task 2				COVID-19 patient deterioration	pH, pO2, pCO2, lactate, c-reactive protein, hematocrit, hemoglobin, WBC, platelet count, ALT, AST, d-dimer, alkaline phosphatase, bilirubin, fibrinogen, albumin, creatinine, sodium, potassium, MCH, MCHC, MCV, red blood cells, RDW, eosinophils, lymphocytes, basophils, monocytes
	Deteriorate	281	1059		
	No-deteriorate	36	2278		
	**Total**	**317**	**3337**		

**Labels.** For the Data4u dataset, we used two labels for prediction. The first label is *COVID-19 diagnosis*, which is encoded as a binary variable where 1 indicates having COVID-19 disease and 0 indicates not having the disease. The second label is *potential patient deterioration*, derived from the *admission to semi-intensive care units* and *admission to intensive care units* features. The label value is 0 if both *admission to semi-intensive care units* and *admission to intensive care units* are 0; otherwise, it is 1. Note that this implies that we do not model the extent of deterioration, only whether the patient’s condition deteriorates or not. This means that patients admitted to the regular ward (but not semi-intensive (high-dependency) or intensive care) are not considered to have a deteriorated condition. This was done to improve the potential of AI/ML algorithms to learn robust dependencies from the data rather than fit on noise. Indeed, the number of patients admitted to semi-intensive or intensive care in the data is relatively small for modeling purposes.

For the MIMIC-IV dataset, we specifically targeted *patient deterioration*. This label was derived from ICU admission data: assigned a value of 1 if the patient was admitted to the ICU during the same hospital stay as the COVID-19 diagnosis, and 0 otherwise. Similar to the Data4u dataset, we considered admission to the ICU as the primary indicator of deterioration, regardless of the duration of stay.

### Missing data handling

The optimal way to handle missing data depends on the mechanism that results in the missing values, with the two main approaches being excluding the records with missing entries and replacing the missing entries using imputation [21]. The decision on whether to impute or not depends on the rate of missing values and the underlying mechanism that causes the missing entries. Generally, missing data can be a result of one of three processes: missing completely at random (MCAR), missing not at random (MNAR), or missing at random (MAR) [22]. MCAR is a result of random errors, such as equipment failures, whereas MNAR occurs when the mechanism causing the missing entries has no discernible patterns. MAR is the most common type of missing data and is defined as missing data that is dependent on observed values. Most of the missing data in our case results from one or more laboratory tests being omitted at the hospital depending on the medical analysis, and thus most of the missing entries in the data considered in this paper are MAR in nature. MCAR data can be deleted without risking adding bias to the results whereas imputation is recommended for missing values of MAR or MNAR types [23].

**Fig 2:**
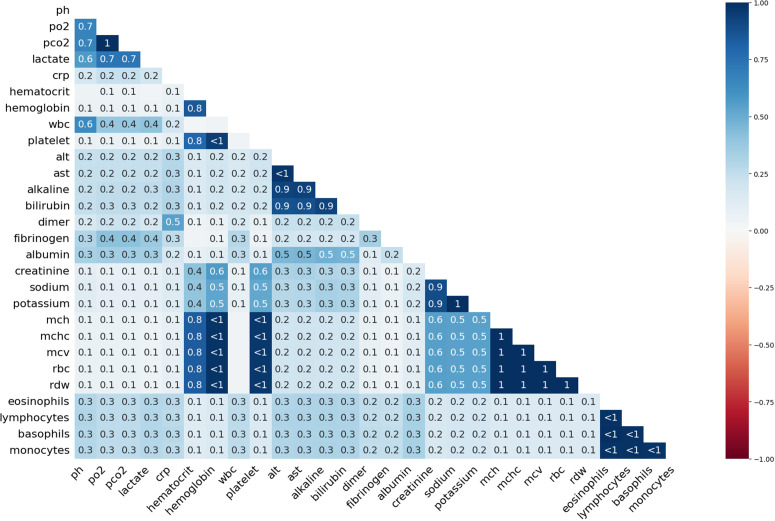
Heatmap of features co-occurrence of missingness (MIMIC-IV dataset).

We separately assess how the profile of missing features affects the performance of imputation techniques and the performance of the AI/ML techniques used for predicting COVID-19 status and outcomes. The profile of missing features is characterized by the composition of the features that contain missing values (categorical, numerical, or mixed), the rate of missing values, and the pattern of the missing features. The pattern refers to the likelihood of data being missed together (togetherness) or independently (randomness). This pattern is motivated by the observation that certain combinations of laboratory tests either miss consistently or are present together. For example, features related to blood characteristics require a sample of blood from the patient and if this sample is not taken then all related features are necessarily missing. We verified the existence of this pattern by performing a pairwise correlation analysis of the presence and absence of features. All feature pairs with perfect correlation were merged into the togetherness pattern whereas others were encoded as random. Fig 2 illustrates the missingness patterns of features and their correlation values for the MIMIC-IV dataset. The subset of features that have very high correlation (more than 90) are considered in one together subset. The patterns are only used for comparing the imputation performance of imputation methods.

We assess six approaches for handling missing values in this study: (i) no imputation (baseline), where records with missing entries are excluded; (ii) single imputation using a random draw from the observed values; (iii) multiple imputation using KNN, which estimates missing entries from the k most similar samples; (iv) multiple imputation using MICE (multiple imputation by chained equations), which iteratively models each variable conditional on the others to generate multiple completed datasets and combines estimates; (v) SoftImpute, a low-rank matrix completion method that leverages global structure via nuclear-norm regularization; and (vi) MissForest, an iterative random-forest imputation method that accommodates mixed data types and nonlinear relationships. KNN and MICE are commonly used methods for imputing data [24] and they have been shown to perform well consistently, particularly for larger datasets [25]. The diversity of these methods allows us to compare various imputation methods and assess accuracy in imputing clinical data and evaluate their impact on ML predictions and clinical implications.

**Fig 3:**
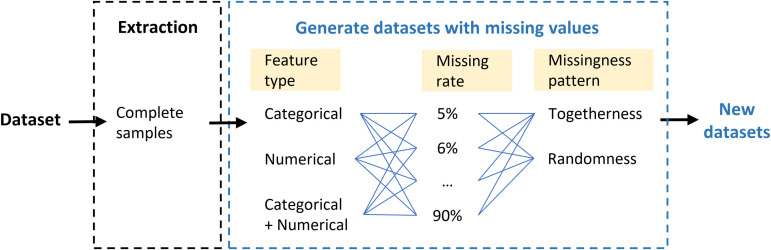
Pipeline generating datasets with different missingness profiles.

The imputation techniques are evaluated using simulated datasets that are realistic but that reflect different profiles of missing data. As a baseline, we consider the complete data, and hence the evaluation is limited to the 362 samples in Data4u dataset and 317 samples in MIMIC-IV COVID dataset that contain no missing values. Our focus on complete records is driven by the need to establish a controlled simulation environment to systematically investigate the impact of various rates of missingness on the performance of ML models. This approach allows us to synthetically inject missingness into the dataset and evaluate the models under different missingness patterns. We create 86 copies of these data by considering different levels of missing rate (5% − 90%, 1% increment) of three feature types (categorical, numerical, categorical+numerical) following the pipeline depicted in Fig 3. Missing rates of 5% and 90% are selected as a lower bound and an upper bound of the missing rate to examine ML models performance under low and extreme missingness. In practice, low missingness rate (e.g., ≈5%) is often considered manageable, whereas very high missingness (e.g., ≈90%) is typically excluded as they cannot be meaningfully imputed due to lacking sufficient observed information to estimate missing values and can severely limit the reliability of imputation and downstream analyses [23,26]. Data is randomly dropped following either the togetherness pattern or the randomness pattern. In total, 516 datasets (3 feature types x 86 missing rates x 2 missingness patterns) were created in total, each represents a unique missingness profile. These datasets are referred to as simulated datasets. The five imputation techniques (single, KNN, MICE, SoftImpute, and MissForest) were run on each simulated dataset 500 times and the mean absolute error (MAE) of the values was computed using Equation 1 and compared to the baseline, i.e., the complete data.


$$\begin{array}{c} MAE=\frac{1}{N}\sum_{i=1}^{N}|V_{i}^{\prime }-V_{i}| \end{array}$$ (1)

where N is the total samples, $V_{i}$ represents actual values and $V_{i}^{\prime }$ denotes the values predicted by imputation. MAE is a widely used, interpretable metric that measures the average absolute error between imputed and true values in the complete dataset to evaluate value-level imputation accuracy. Moreover, MAE is more robust to large outliers compared to other metrics such as RMSE, thus provides a conservative assessment of average errors across repeated imputations [27].

### Infection and patient deterioration prediction

Classifiers were trained and tested for detecting COVID-19 infections and predicting patient deterioration from laboratory tests. As described in the previous section, for the Data4u dataset, the targets were encoded as positive (1) or negative (0) depending on the value of the RT-PCR test (Task 1) or whether the patient was admitted to semi-intensive ward or intensive care (Task 2). Both tasks were carried out on two datasets, one where all entries with missing values were excluded (i.e., complete) and one where entries with missing values were included; see Table 1. The latter was imputed using MICE and SoftImpute for Data4u and MIMIC-IV, respectively as they yielded the lowest error in clinical settings for corresponding datasets; see Section Imputation performance.

For data from the MIMIC-IV dataset, we only have Task 2 and the target was encoded as patient admitted to intensive care (1) or not (0).

Six different classification algorithms were considered: logistic regression (LR), random forest (RF), support vector machine (SVM), k-nearest neighbors (KNN), XGBoost, and LightGBM models. These six learning models were selected for their performance on tabular data [28] and offering a degree of interpretability with feature ascription methods [29]. Deep learning was omitted due to the number of entries being insufficient for reliably validating overfitting.

**Evaluation metrics.** The performance of the learning models is compared using accuracy, balanced accuracy, sensitivity, specificity, and the area under the curve (AUC). Sensitivity and specificity represent the percentage of individuals being correctly identified as positive or negative for the condition of interest, i.e., COVID-19 infection or patient deterioration, respectively. Sensitivity determines whether the test is satisfactory compared to a reference standard whereas specificity is important in establishing a patient tested positive on the screening test do or do not actually possess the condition [30]. Since the proposed imputation and AI/ML pipeline emphasizes both aspects, we calculated the average of sensitivity and specificity scores, i.e., balanced accuracy [13], and included it as a performance metric. Lastly, AUC depicts the degree of separability between classified classes, i.e., patients with and without COVID-19, or patients being admitted to semi-/intensive care units or not.

The training and test data were obtained using a stratified 5-fold cross-validation technique to balance the bias and variance of the small dataset during training. To address the imbalanced class problem, the synthetic minority oversampling technique (SMOTE) was used to increase the minority class samples in the training dataset so that the model can generalize well on new unseen data. After applying SMOTE method, the dataset become more balanced to differentiate between the target classes.

To understand the impact of features on the predictive performance of the different models, we used Shapley Additive Explanations (SHAP) to estimate the average marginal contribution of each feature and used this to assess the importance of different features. The Shapley value originates from coalitional game theory and is a popular method for estimating the overall contribution of a feature for a given prediction [31]. The higher the absolute Shapley value of a feature, the more significant the contribution of a feature is on the predictions given by a model. The contributions of features can be visualized using a summary plot where the x-axis corresponds to the Shapley values and the y-axis to the different features.

### Implementation

All algorithms and experiments were implemented in Python (version 3.8). NumPy [32] was used for numerical data processing, Pandas [33] for data processing, the scikit-learn library [34] for data processing, imputation and the classifier implementation, MissForest for MissForest imputation, fancyimpute for SoftImpute imputation, and Matplotlib [35] for data visualization. We used the default hyperparameters of the scikit-learn library for our ML methods, as our goal was to investigate the effect of various imputation methods on the ML model performance. S1 Table in the Supporting information shows the hyperparameters of the ML methods used in this study.

## Results

In this section, we first assess the performance of imputation methods across different patterns and rates of missingness. We then analyze the performance of commonly used machine learning models on both complete and imputed datasets for infection prediction and patient deterioration prediction, evaluating accuracy, balanced accuracy, sensitivity, specificity, and AUC. Finally, we investigate the important features for the best performing models in infection prediction and patient deterioration prediction using Shapley values.

### Imputation performance

In this section, we compare the performance of various imputation methods for different rates of missing for both of our datasets.

**Fig 4:**
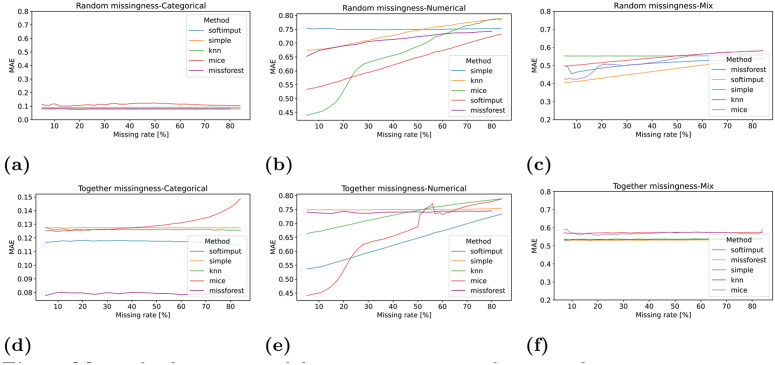
Mean absolute errors of data imputation in relation with missing rates (5%–90%) and missingness patterns (randomness: a-c, togetherness: d-f) (The average CI for all the plots were smaller than [−0.01, 0.01] and they are not shown in this figure.).

**Einstein Data4u.** Fig 4 depicts the Mean Absolute Errors (MAE) of data imputation using simple imputation, multiple imputation KNN, multiple imputation MICE, SoftImpute, and MissForest at 86 levels of missing rates (from 5% to 90% at 1% increments) of different feature types and patterns of missing data, following the simulated data generation pipeline detailed in Section Missing data handling. 95% Confidence Intervals (CIs) for these MAE values were computed and are reported as part of the figures.

As seen from the figure, imputation using MICE yields the lowest errors compared to KNN and simple imputation techniques for typical clinical scenarios (missingness < 25%). However, the performance landscape shifts at higher sparsity levels. While MICE is dominant in low-missingness numerical settings, SoftImpute closely follows MICE for these rates and eventually outperforms all other methods as missingness increases beyond 30%.

Simple imputation, SoftImpute, and MissForest are best suited for missing categorical data or data having a high percentage of missing numerical data that are correlated. KNN imputation works better than simple imputation when the missing numerical data is uncorrelated, i.e., the values are randomly missing across multiple rows. When the missing data pattern is random, multiple imputation using MICE yields the lowest error for numerical data. For higher sparsity, and more complex settings, such as categorical or mixed features, MICE remains competitive but techniques like SoftImpute and MissForest often yield the strongest performance.

**Fig 5:**
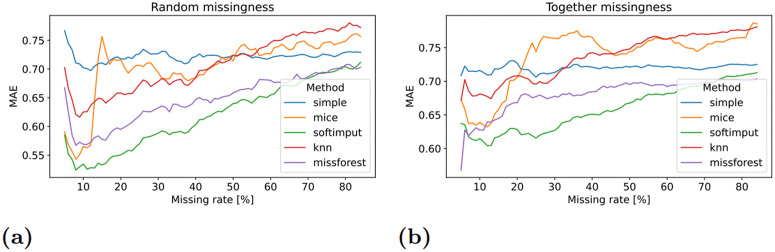
Mean absolute errors of data imputation in relation with missing rates (5%–90%) and missingness patterns for MIMIC-IV dataset (randomness: a, togetherness: b) (The average CI for all the plots were smaller than [−0.01, 0.01] and they are not shown in this figure.).

**MIMIC-IV.** Fig 5 demonstrates the performance of imputation methods on the MIMIC-IV dataset. As shown in the figure, for this data, SoftImpute consistently outperforms other methods, followed by MissForest. SoftImpute is a matrix-completion method that leverages the global covariance structure of the data via nuclear-norm regularization, unlike KNN which relies on local neighborhoods. This characteristic allows SoftImpute to effectively capture inherent relationships between features even at high rates of missingness. MissForest, being based on random forests, captures non-linear complex relationships between features but is computationally more intensive. Simple imputation ignores feature correlations and thus yields higher error. As missingness increases, the performance of KNN and MICE degrades more rapidly than SoftImpute, suggesting they are less robust to extreme sparsity in this specific dataset and that the best performing methods can depend on the data characteristics, as well as specifics of the processing performed on the data.

**General Findings.** When analyzed across both datasets, most imputation techniques target data that is missing at random (MAR), and both single and multiple imputation also fare best in these cases with our data. KNN imputation seems best suited for data that is missing completely at random (MCAR). Overall, the results indicate that imputation is important for increasing data coverage, but that it is also important to assess the patterns of missing data and achieving the best possible result may require combining different imputation strategies.

**Statistical Validation.** To verify the statistical significance of the main findings, we conducted pairwise statistical comparisons using Welch’s two-sample t-test on the reconstruction errors (MAE) across the 500 simulation folds. For the Einstein Data4u cohort, advanced multivariate methods (MICE and SoftImpute) consistently yielded significantly lower errors (*p* < 0.05) than baseline methods across low and moderate missingness rates. As expected, at extreme levels of data sparsity (typically exceeding 50% to 60%), the performance gap began to narrow, with differences occasionally becoming statistically non-significant (p≥0.05). Under the “together” missingness pattern, this convergence usually occurred at lower sparsity thresholds than under the random missingness pattern. This convergence reflects the theoretical limits of imputation. When the majority of data or larger blocks of related clinical features are missing, underlying joint distributions cannot be reliably modeled, causing advanced techniques to perform similarly to simpler baselines. However, within clinically realistic sparsity ranges, the statistical advantage of multivariate methods remained robust. For the MIMIC-IV dataset, predictive differences between multivariate and univariate imputation consistently demonstrated statistical significance (*p* < 0.05) across all evaluated missingness rates, with a single exception (KNN vs. MissForest at 22% missingness, *p* = 0.166).

### Prediction of COVID-19 infections and patient deterioration

The predictive performance for COVID-19 infections and patient deterioration on the Einstein Data4u dataset is summarized in Table 3. The performance was assessed by comparing predictions from a complete dataset (where records lacking any laboratory test were removed) to those from a dataset imputed using MICE. As shown in Table 3, for the Data4u dataset, all models demonstrate high accuracy and balanced accuracy, reaching up to 97% and 90% for COVID-19 diagnosis, and up to 96% and 82% for predicting patient deterioration. The consistent improvement of these performance metrics on the imputed data demonstrates the effectiveness of the proposed pipeline.

**Table 3:** Results for predicting COVID-19 infection and for predicting patient deterioration (Data4u Dataset). The best value for each score is denoted in bold.

Dataset	Prediction	LR	RF	SVM	KNN	XGBoost	LightGBM	Average
Complete	**COVID-19 diagnosis**							
	Accuracy	0.91	0.91	0.90	**0.93**	0.88	0.89	0.90
	Balanced accuracy	**0.80**	0.78	0.66	0.79	0.71	0.73	0.74
	Sensitivity	0.64	0.59	0.32	0.59	0.69	**0.71**	0.59
	Specificity	0.95	0.96	**0.99**	**0.99**	0.95	0.97	0.97
	AUC	0.92	0.92	0.88	**0.94**	0.87	0.86	0.90
	**Patient deterioration**							
	Accuracy	0.83	0.85	**0.86**	0.85	0.85	0.84	0.85
	Balanced accuracy	0.74	0.78	**0.79**	0.77	0.65	0.64	0.73
	Sensitivity	0.53	0.61	0.61	0.54	**0.63**	**0.63**	0.59
	Specificity	0.96	0.96	0.97	**0.99**	0.95	0.94	0.96
	AUC	0.69	0.74	**0.84**	0.78	0.83	0.83	0.78
Incomplete dataset with MICE Imputation	**COVID-19 diagnosis**							
	Accuracy	0.96	**0.97**	0.96	**0.97**	0.92	0.93	0.95
	Balanced accuracy	0.85	**0.90**	0.79	0.86	0.76	0.79	0.82
	Sensitivity	0.71	**0.81**	0.58	0.72	0.74	0.76	0.72
	Specificity	0.98	0.98	**0.99**	**0.99**	0.96	0.98	0.98
	AUC	0.96	0.98	0.97	**0.99**	0.89	0.90	0.95
	**Patient deterioration**							
	Accuracy	0.95	0.95	**0.96**	0.95	0.89	0.89	0.95
	Balanced accuracy	0.81	0.80	**0.82**	0.76	0.71	0.70	0.77
	Sensitivity	0.62	0.61	0.65	0.53	**0.68**	**0.68**	0.63
	Specificity	**0.99**	**0.99**	**0.99**	**0.99**	0.96	0.97	0.98
	AUC	0.79	0.84	**0.94**	0.90	0.91	0.88	0.88

**Infection detection.** Overall, for the incomplete dataset, the probability of correctly predicting non-infected COVID-19 cases (specificity) is between 96% and 99% across models, and the probability of correctly predicting COVID-19 infections (sensitivity) reaches up to 81%. While specificity remains comparable across datasets, the sensitivity is significantly lower on the complete dataset. All models show strong predictive performance on the incomplete dataset. Minimum values for accuracy, balanced accuracy, and AUC are 0.92, 0.76, and 0.89, respectively. On the complete dataset, these baselines drop to 0.88 (accuracy), 0.66 (balanced accuracy), and 0.86 (AUC).

In terms of algorithms, RF and KNN achieve the best performance, followed closely by SVM and LR. XGBoost and LightGBM also perform well, though their AUCs are comparatively lower than RF or KNN for this specific task. More importantly, the results show a high improvement in correctly detecting COVID-19 infection cases (sensitivity) when using the imputed dataset. RF shows a 22% increase in sensitivity compared to the complete case, while SVM shows a 26% increase. These results suggest that patients testing positive with our pipeline are highly likely to be infected, allowing for the prioritization of RT-PCR testing and providing a simple method to identify early infections prior to symptom onset.

**Patient deterioration prediction.** As shown in Table 3, SVM is the best performing model for predicting patient deterioration, followed by LR, RF, and KNN. LightGBM and XGBoost also achieve high specificity and AUC. However, their balanced accuracies are lower than the other models. The sensitivity score increases by 4% with SVM (sensitivity = 0.65), by 5% with LightGBM and XGBoost (sensitivity = 0.68), and by 9% with LR (sensitivity = 0.62) when prediction is performed on the imputed dataset. Failing to detect the need for semi-/intensive care units for 9% of patients can rapidly burden the healthcare system and, in the worst case, lead to loss of life. While the sensitivity of the KNN and RF models remains practically unchanged, their AUC scores increase when data is imputed, indicating a better ability to distinguish patients likely to deteriorate. The specificity of all models is very high (minimum 0.94) regardless of whether the data is imputed or not. In other words, any positive prediction for deterioration should be treated as a high-priority admission to semi-/intensive care, as the likelihood of false positives is minimal. This highlights the potential of using our approach for triaging COVID-19 patients based on simpler laboratory tests.

**Patient deterioration prediction on MIMIC-IV dataset.** On the MIMIC-IV COVID cohort, the trend reverses compared to the Data4u dataset. Models trained and evaluated on the complete dataset consistently outperform models trained on incomplete datasets with imputed data (Table 4). RF achieves the highest performance on complete cases, with AUC, balanced accuracy, and sensitivity values of 0.99, 0.96, and 0.98, respectively. The performance improvements, however, should be interpreted with caution as they suggest that the missingness mechanism in MIMIC-IV is Informative (Missing Not At Random – MNAR) or that the complete dataset represents a biased sub-population. Indeed, as shown in Table 4, the complete dataset contains only 36 records of the non-deteriorate class, whereas in the incomplete dataset, the non-deteriorate class is twice the size of the deteriorate class. This implies that complete case analysis filters for a specific subset of patients (likely those heavily monitored due to severity), introducing selection bias. While imputation yields lower raw metrics than this (biased) baseline, it allows analyzing broader and more representative patient cohort. These results emphasize that while imputation is beneficial for increasing coverage, its impact on predictive metrics depends heavily on the underlying missingness mechanism (MAR vs. MNAR) and population characteristics.

**Table 4:** Results for predicting patient deterioration (MIMIC-IV COVID Dataset). The best value for each score is denoted in bold.

Dataset	Prediction	LR	RF	SVM	KNN	XGBoost	LightGBM	Average
Complete	Accuracy	0.7904	**0.9623**	0.9266	0.7987	0.9308	0.9497	0.8930
	Balanced accuracy	0.7873	**0.9579**	0.9269	0.8238	0.9289	0.9480	0.8954
	Sensitivity	0.8043	**0.9822**	0.9253	0.6833	0.9395	0.9572	0.8819
	Specificity	0.7704	0.9337	0.9286	**0.9643**	0.9184	0.9387	0.9090
	AUC	0.8672	**0.9922**	0.9813	0.9370	0.9867	0.9891	0.9589
Incomplete dataset with MICE Imputation	Accuracy	0.8142	**0.8529**	0.8364	0.8121	**0.8541**	**0.8541**	0.8373
	Balanced accuracy	0.7694	**0.8296**	0.8051	0.7550	0.8267	0.8272	0.8022
	Sensitivity	0.6468	**0.7658**	0.7195	0.5987	0.7516	0.7535	0.7060
	Specificity	0.8920	0.8933	0.8907	**0.9113**	0.9017	0.9008	0.8983
	AUC	0.8621	**0.9021**	0.8824	0.8390	0.8957	**0.9023**	0.8806
Incomplete dataset with SoftImpute	Accuracy	0.8316	0.8621	0.8358	0.8157	0.8613	**0.9497**	0.8644
	Balanced accuracy	0.7962	**0.8396**	0.7989	0.7681	0.8361	0.8373	0.8127
	Sensitivity	0.6996	**0.7770**	0.6973	0.6833	0.7659	0.7632	0.7311
	Specificity	0.8929	0.9022	0.9004	0.8986	0.9062	**0.9115**	0.9020
	AUC	**0.9061**	0.8918	0.8557	0.9021	**0.9072**	0.8917	0.8924

### Feature importance

We analyzed feature importance using Shapley Additive Explanations (SHAP) to understand how imputation affects the clinical factors driving model predictions.

**Fig 6:**
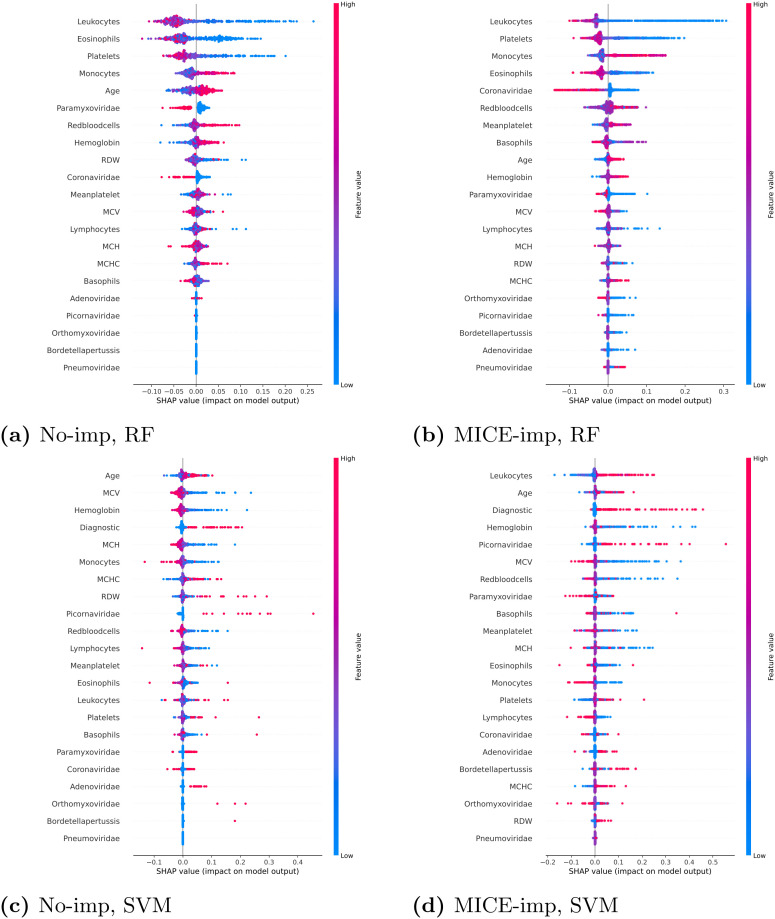
Impact of the features on predicting COVID-19 diagnosis (a, b) and patient deterioration (c, d).

**Data4u Dataset.** The feature importance for predicting COVID-19 diagnosis using the best-performing model (RF) is shown for the complete and imputed datasets in Fig 6a and 6b, respectively. Similarly, Fig 6c and 6d display the feature importance for predicting patient deterioration using the best-performing model (SVM). In these SHAP summary plots, each horizontal bar corresponds to a specific feature. The length of the bar indicates the magnitude of the SHAP value (impact on prediction), while the color represents the feature value (red for high, blue for low).

For COVID-19 diagnosis (Figs 6a, b), the feature ranking is consistent across both complete and imputed datasets. Blood variables—specifically leukocytes, platelets, monocytes, and eosinophils—consistently emerge as the most critical indicators for detecting the presence of the virus. For patient deterioration (Figs 6c, d), imputation brings subtle but important shifts. While key inflammatory markers remain important, the imputed model (SVM) places greater weight on a broader set of viral family coinfections compared to the complete-case model, suggesting that imputation recovers signals from sparser viral panel data.

**Fig 7:**
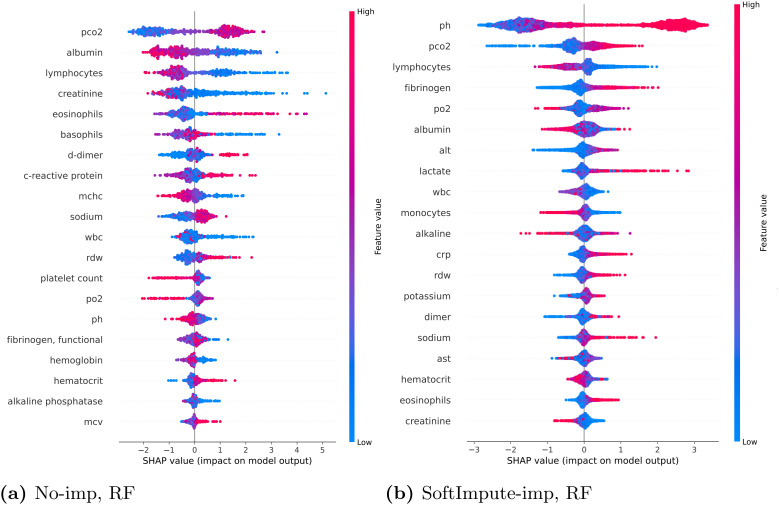
Impact of the features on predicting patient deterioration for MIMIC-IV dataset.

**MIMIC-IV Dataset.** Fig 7 illustrates the shift in feature importance for patient deterioration in the MIMIC-IV cohort. The model trained on the complete dataset (Fig 7a) relies heavily on pCO2 (partial pressure of carbon dioxide) and specific blood inflammation parameters. While highly predictive for the subset of patients with these tests, this view is limited. In contrast, the model trained on the imputed dataset (Fig 7b) reveals a more comprehensive clinical picture. Indicators of lung function (pH, pO2, pCO2) alongside broader markers of inflammation and immunity emerge as the top predictors. This demonstrates that even when imputation does not drastically improve predictive metrics (as seen in the previous section), it fundamentally alters the clinical narrative. By enabling the inclusion of patients with partial data, imputation reduces selection bias and unmasks risk factors related to respiratory failure that are obscured in the smaller, complete-case cohort.

## Discussion

### Imputation and model performance

Handling missing data in clinical datasets is a significant challenge, as excluding records with missing values can introduce biases and lead to models with poor generalizability. Our study systematically investigates the effects of various imputation methods on AI/ML model performance, demonstrating that the choice of imputation strategy significantly influences the performance of ML models for detecting COVID-19 infection cases or patient deterioration. Specifically, we found that a lower number of infection cases are detected when missing values are excluded, which could result in many patients requiring admission to semi-intensive (high-dependency) or intensive care units being overlooked.

Our results on the Einstein Data4u dataset indicate that the accuracy, balanced accuracy, sensitivity, specificity, and AUC of different models for predicting both COVID-19 infections and patient deterioration are distinctly higher when using imputed data compared to a complete dataset. Conversely, for the MIMIC-IV dataset, the absolute performance metrics are numerically higher with the complete dataset, which is consistent with selection bias. The complete subset in MIMIC-IV represents a distinct, heavily monitored patient population (where tests are ordered due to clinical severity), whereas the imputed dataset captures the broader, more representative cohort. This demonstrates that complete-case analysis may yield higher apparent accuracy but is limited to a biased sub-population; in contrast, imputation enables decision-making for the general patient population, even if the inclusion of noisier data results in more conservative raw metrics.

In our experiments, multivariate imputation methods (MICE and SoftImpute) resulted both in the lowest reconstruction error (MAE) and the highest sensitivity. A lower MAE indicates that the imputed values closely mirror the true underlying clinical data. By minimizing the absolute error during reconstruction, multivariate methods like MICE and SoftImpute better preserve the natural distributions, variance, and multivariate correlations present in laboratory tests. Consequently, the link between lower MAE and predictive utility stems from highly accurate data reconstruction, which is essential for ensemble tree-based classifiers (Random Forest, XGBoost) that rely on the continuous data spread to identify optimal decision boundaries as destroying this variance prevents effective class separation. The same dependency extends to models using hyperplanes or distance metrics (SVM, KNN), where artificial mean-clustering skews the feature space topology.

### Clinical Implications

The clinical implications of our findings are central to the operational utility of healthcare AI. For providers, our study provides a framework for selecting imputation methods that maximize diagnostic sensitivity—the primary requirement for early screening. Policymakers can leverage these results to inform public health strategies, enabling timely intervention in resource-limited settings where RT-PCR availability is constrained.

The AI/ML models for detecting infections leverage established laboratory procedures instead of requiring specialized tests, facilitating timely interventions. Early population screening using laboratory tests enables medical authorities to understand disease prevalence and severity, allowing for effective prioritization of public health interventions. Additionally, demand prediction helps identify the needs of different hospital wards, alerting authorities to potential shortages of semi-intensive or intensive care unit beds in advance.

### Feature importance and its variability

The analysis of feature importance indicated that certain features, such as blood variables (leukocytes, platelets, monocytes, eosinophils), consistently play a crucial role in detecting COVID-19 infections. However, the importance of some features varies significantly depending on whether the data are imputed. For example, high levels of Pneumoviridae or low levels of Bordetella pertussis indicate a likely infection when the data are imputed, but these features do not contribute to prediction accuracy when trained on the complete dataset.

Similarly, in predicting patient deterioration, the significance of features changes after imputation. For the Einstein Data4u dataset, high values of leukocytes and Picornaviridae indicate a higher likelihood of deterioration, which is less apparent in the complete dataset. In the MIMIC-IV dataset, markers related to respiratory function (e.g., pH and pO2) showed higher importance in the imputed dataset compared to the complete dataset, suggesting that restricting analysis to complete cases may obscure critical respiratory signals present in the broader population.

### Comparison with previous work

While previous studies have established the feasibility of COVID-19 screening using laboratory data, they often present a static view of clinical predictors based on complete-case analysis or simple imputation. For example, a logistic regression model using complete blood count data and sex was proposed by Joshi et al. to predict SARS-CoV-2 PCR positivity and improve testing resource allocation [14]. In turn, Brinati et al. showed that machine learning models based on routine blood tests can achieve performance comparable to rRT-PCR while enabling interpretable and accessible COVID-19 screening tools [13]. Our work extends these findings by demonstrating that clinical insights are tied to the imputation strategy employed. Specifically, while foundational models commonly highlight leukocytes and platelets as top predictors – a finding we also replicate – our systematic approach reveals that the inclusion of imputed viral panel data unmasks additional predictors (e.g., specific pathogen coinfections) that are entirely obscured in complete-case cohorts.

In addition, our comparison between the Data4u and MIMIC-IV datasets provides insights into performance differences between complete-case and imputed analyses. Although imputation is often recommended to enhance performance, generalizability, and reduce bias, these benefits depend on the plausibility of the assumed missing data mechanism (e.g., MAR) and correct model specification. Our results on the MIMIC-IV cohort indicate that, in intensive care settings, higher apparent predictive metrics in complete cases may reflect selection bias. By shifting emphasis from pure accuracy to the validity and transportability of the clinical narrative, we offer a more realistic framework for deploying AI/ML in clinical environments, where data are seldom missing at random.

### Limitations and future work

While our study provides valuable insights, it is not without limitations. First, the presented pipeline aims to provide valid analytic estimation in the presence of missing data while preserving relationships among features, rather than estimating the precise value for each variable. Second, we focused on classical and ensemble machine learning models (RF, SVM, XGBoost) and did not include deep learning architectures. This was a deliberate choice driven by the limited sample size of the primary cohort, where deep models are prone to overfitting.

Third, our patient deterioration target combines admissions to semi-intensive and intensive care units. While necessary to achieve sufficient sample sizes for robust machine learning, we acknowledge that this binarization oversimplifies clinically heterogeneous outcomes. Patients may require intensive care for a numerous of physiological reasons, e.g., isolated respiratory failure versus systemic septic shock, and the use of a binary target loses information on time-to-event dynamics. At the same time, this abstraction does not invalidate our methodological conclusions. The demonstrated capability of multivariate imputation to reconstruct underlying data distributions and recover latent prognostic signals from sparse laboratory tests remains robust, regardless of the target’s clinical heterogeneity. Nevertheless, our pipeline should be viewed primarily as an early-warning triage tool rather than a granular physiological tracker.

Fourth, our simulation study relies on MAR/MCAR assumptions. While the comparison with MIMIC-IV highlights the impact of informative missingness (MNAR), synthetic metrics cannot fully capture the complexity of real-world clinical bias where test ordering is tied to unobserved health states. Finally, the results are based on specific geographic regions and clinical protocols. Future work should validate this approach on larger, multi-center datasets to further assess generalizability across diverse patient populations.

### Ethical considerations

Imputation in biomedical research introduces ethical concerns about algorithmic fairness and transparency. If imputation models are trained on datasets that underrepresent specific demographics, they may systematically introduce biases that lead to inequitable clinical decisions for those groups. Similarly, automated data filling can create a false sense of certainty in clinical high-stakes scenarios. To mitigate these risks, our pipeline utilizes Explainable AI (SHAP) methods to ensure that imputed predictors are physiologically plausible and transparent to the clinician. We argue that imputation should be viewed as a tool to improve population-level representation rather than a replacement for primary data collection, and its deployment must be accompanied by rigorous bias auditing.

### Guidelines

The results demonstrated that both missing data and the selection of imputation techniques for handling it affect the performance of AI/ML models trained on patient records. In the following, we provide a summary of the key guidelines on how to best handle missing data to improve the quality of the data and AI/ML model performance.

**Step 1. Pruning the features.**

- *Feature-demand filtering:* The first step is to understand the nature and reason for missing data and to classify features with missing values as Missing Completely at Random (MCAR), Missing Not at Random (MNAR), or Missing at Random (MAR). The features classified as MCAR can be omitted without the risk of adding bias [25]. For features classified as MAR, those having missing rates between 5% and 90% should be retained. However, caution is needed for MNAR features (e.g., tests not ordered for healthy patients); excluding them may remove valuable clinical signals, as seen in our MIMIC-IV analysis.
- *Feature-relatedness filtering:* Dependencies between features can degrade imputation performance and hence it is important to identify features (i.e., test procedures) that are closely related and to merge them. This can be done using hierarchical clustering to produce a clinically meaningful division of the data. Crucially, this step must be performed either using established domain knowledge (e.g., viral taxonomy) or nested within the cross-validation loop to prevent data leakage from the test set.
- *Feature-redundancy filtering:* Features that are highly correlated among one another contain significant redundancy and can again degrade imputation performance. As a result, only one of the redundant features should be selected. For example, in the case of blood tests, the information of the red blood cells can be obtained from hematocrit and hemoglobin test results, so selecting the one with the better feature profile suffices.

**Step 2. Identifying the missingness profile.** Features should be associated with a missingness profile that comprises the composition of the features that contain missing values, the rate of missing values, and the pattern of the missing features. In the context of personal medical records, the missingness profile can be determined based on the medical practices and procedures that govern which medical tests and procedures a specific patient undergoes.

**Step 3. Identifying the best imputation technique(s).** Imputation generally improves performance, but the imputation technique should be chosen based on the missingness profile identified in Step 2.

- *Low to Moderate Missingness (< 25%):* For datasets with typical clinical missingness rates, **MICE** yields the lowest error and is the recommended default for tabular medical data.
- *High Missingness (> 30%):* For datasets with high sparsity, matrix-completion methods like **SoftImpute** are more robust than iterative regression methods. They leverage global covariance structures to recover signals where local neighbors (KNN) or regression targets (MICE) become unreliable.
- *Mixed Data Types:* For datasets with complex non-linear relationships or mixed categorical/numerical data, tree-based methods like **MissForest** offer a strong alternative, though at a higher computational cost.

## Conclusion

We systematically analyzed the effects of various imputation methods on the performance of AI/ML techniques, emphasizing the critical role of missing data handling in deriving accurate clinical insights. Unlike previous studies that often default to simple mean imputation or complete-case analysis, our work quantitatively demonstrates that the choice of imputation strategy is not merely a technical preprocessing step but a clinical decision that fundamentally alters risk profiling.

Through a comprehensive evaluation on two distinct cohorts—the Einstein Data4u dataset (screening) and the MIMIC-IV dataset (intensive care)—we reveal a dichotomy in imputation effects. For general screening populations (Data4u), appropriate imputation (specifically MICE) can improve sensitivity by up to 26 percentage points, enabling the recovery of critical diagnostic signals from sparse data. Conversely, in intensive care settings (MIMIC-IV), while imputation may not exceed the raw predictive metrics of complete-case analysis due to informative missingness, it plays a vital role in reducing selection bias. By enabling the inclusion of patients with partial data, imputation unmasks broader risk factors (e.g., respiratory markers) that are otherwise hidden in heavily monitored sub-populations.

We benchmarked six common classification algorithms and six imputation strategies, finding that tree-based models (Random Forest, XGBoost) combined with multivariate imputation (MICE or SoftImpute) offer the most robust performance for tabular clinical data. These findings underscore that optimal imputation is context-dependent: MICE excels in low-missingness numerical scenarios, while matrix-factorization methods like SoftImpute are superior for high-sparsity regimes.

Beyond providing guidelines, our work illustrates that effective handling of missing values is essential for robust AI/ML applications in healthcare, particularly for systems in resource-limited settings where data sparsity is high. While limited by the sample size of the primary cohort preventing the use of deep learning architectures, our study provides a validated, cost-effective pipeline for early COVID-19 screening and triage. Ultimately, our research contributes to the advancement of reliable clinical AI, ensuring that data processing choices lead to transparent, accurate, and unbiased patient outcomes.

## Supporting information

S1 Table ML models’ Hyperparameters. (PDF)
