# Supplementary material for: Mission imputable: Effects of missing data processing on infectious disease detection and prognosis
Source: PLoS One. 2026 Jul 27;21(7):e0320105. doi: 10.1371/journal.pone.0320105 (PMC13405099; doi:10.1371/journal.pone.0320105)
Supplement: S1 Table — (PDF) [file pone.0320105.s001.pdf]

S1 Table. ML models' Hyperparameters

Machine Learning Model	Parameter Name	Value of the Parameter
**Logistic Regression**	penalty	'l2'
	C	1.0
	solver	'lbfgs'
	max_iter	100
	tol	$1e-4$
	random_state	None
**K-Nearest Neighbors**	n_neighbors	5
	weights	'uniform'
	algorithm	'auto'
	leaf_size	30
	p (Minkowski metric)	2
	metric	'minkowski'
**Support Vector Machine**	C	1.0
	kernel	'rbf'
	degree	3
	gamma	'scale'
	shrinking	True
	probability	False
**Random Forest**	n_estimators	100
	criterion	'gini'
	max_depth	None
	min_samples_split	2
	min_samples_leaf	1
	max_features	'sqrt'
	bootstrap	True
**XGBoost**	objective	'binary:logistic'
	learning_rate (eta)	0.3
	max_depth	6
	gamma	0
	min_child_weight	1
	subsample	1
	colsample_bytree	1
	n_estimators	100
**LightGBM**	objective	'binary'
	learning_rate	0.1
	n_estimators	100
	num_leaves	31
	max_depth	-1
	min_child_samples	20
	subsample	1.0
	colsample_bytree	1.0
